# Beyond the hippocampus: Limbic white matter injury implicated in post-radiation memory performance in primary brain tumor patients

**DOI:** 10.1093/neuonc/noaf144

**Published:** 2025-06-13

**Authors:** Alexander S Qian, Roshan Karunamuni, Soumya Unnikrishnan, Mia A Salans, Austin Hopper, Suma Gudipati, Justin Yu, Michael Connor, Kathryn R Tringale, Michelle D Tibbs, Anny Reyes, Jiwandeep S Kohli, Alena Stasenko, Carrie R McDonald, Jona A Hattangadi-Gluth

**Affiliations:** Department of Radiation Medicine and Applied Sciences, University of California San Diego, La Jolla, California, USA; Department of Radiation Medicine and Applied Sciences, University of California San Diego, La Jolla, California, USA; Department of Radiation Medicine and Applied Sciences, University of California San Diego, La Jolla, California, USA; Department of Radiation Medicine and Applied Sciences, University of California San Diego, La Jolla, California, USA; Department of Radiation Medicine and Applied Sciences, University of California San Diego, La Jolla, California, USA; Department of Radiation Medicine and Applied Sciences, University of California San Diego, La Jolla, California, USA; Department of Radiation Medicine and Applied Sciences, University of California San Diego, La Jolla, California, USA; Department of Radiation Medicine and Applied Sciences, University of California San Diego, La Jolla, California, USA; Department of Radiation Medicine and Applied Sciences, University of California San Diego, La Jolla, California, USA; Department of Radiation Medicine and Applied Sciences, University of California San Diego, La Jolla, California, USA; Department of Psychiatry, University of California San Diego, La Jolla, California, USA; Department of Psychiatry, University of California San Diego, La Jolla, California, USA; Department of Psychiatry, University of California San Diego, La Jolla, California, USA; Department of Psychiatry, University of California San Diego, La Jolla, California, USA; Department of Radiation Medicine and Applied Sciences, University of California San Diego, La Jolla, California, USA; Department of Radiation Medicine and Applied Sciences, University of California San Diego, La Jolla, California, USA

**Keywords:** cingulum, fornix, hippocampal sparing, memory, radiation

## Abstract

**Background:**

Limbic white matter (WM) of the Papez circuit, including the fornix, dorsal cingulum, and parahippocampal cingulum (PHC), interplay with the hippocampus as key components of the memory network. We analyzed biomarkers of injury to these pathways to understand their impact on post-radiation therapy (RT) memory performance.

**Methods:**

Primary brain tumor patients on a prospective trial receiving fractionated brain RT (*n* = 57) underwent volumetric MRI, diffusion tensor imaging, and memory assessments (Hopkins Verbal Learning Test-Revised [HVLT-R] Total and Delayed Recall and Brief Visuospatial Memory Test -Revised [BVMT-R] Total and Delayed Recall) at baseline and 3, 6, and 12 months post-RT. MRI biomarkers included volume, fractional anisotropy (FA), and mean diffusivity (MD). Linear mixed-effects models assessed associations between biomarkers and memory performance over time.

**Results:**

Smaller volumes in the right fornix were associated with lower BVMT-R-Total scores (*P* = 0.019) and left PHC volume loss was associated with worse performance on BVMT-R-Delayed (*P* = 0.039). Lower FA in the left (*P* = 0.010) and right (*P* = 0.019) fornix was associated with lower BVMT-R-Total performance. Lower FA in the left dorsal cingulum (*P* = 0.038) and right PHC (*P = *0.039) were associated with lower HVLT-R-Total and HVLT-R-Delayed scores, respectively. Higher MD in bilateral fornix (*P* = 0.01) and right PHC (*P* = 0.011) correlated with lower BVMT-R-Total scores; higher MD in the right PHC (*P* = 0.046) also correlated with lower HVLT-R-Total scores. Hippocampal volume was not associated with memory scores.

**Conclusion:**

Poorer microstructural integrity in limbic WM tracts of the Papez circuit predicted worse memory performance, while hippocampal injury did not. Dose avoidance in these tracts may preserve memory outcomes.

**Key Points**

• Microstructural injury to limbic white matter tracts such as the fornix predicted worse verbal and visuospatial memory post-RT.
• Hippocampal atrophy was not associated with memory scores.
• Dose avoidance in these areas may improve memory outcomes.

**Importance of the Study**

The study explores the impact of radiation therapy (RT) on memory-related eloquent brain structures beyond the hippocampus, focusing on the Papez circuit’s limbic white matter tracts such as the fornix, dorsal cingulum, and parahippocampal cingulum. While previous research mainly emphasizes hippocampal sparing, this study delves into the effects on efferent and afferent WM tracts like the fornix and cingulum. Using diffusion tensor imaging (DTI), we found RT-induced injury in these tracts correlated with verbal and visuospatial memory decline. Notably, hippocampal atrophy alone didn’t correlate with memory decline, challenging the sole reliance on hippocampal sparing and emphasizing the importance of considering broader memory networks when tailoring RT. The study suggests incorporating WM tract preservation alongside hippocampal sparing for better memory outcomes post-RT, offering insights into refining memory-sparing RT techniques.

Radiotherapy (RT) is a cornerstone in the management of primary and metastatic brain tumors. Unfortunately, it can result in progressive, long-term neurocognitive decline,^1^ which can have devastating effects on post-treatment quality of life.^2,3^ While these neurocognitive changes encompass multiple cognitive domains, including language, processing speed, and executive functioning, memory impairment has received the most attention within the field of neuro-oncology. Indeed, memory deficits are common, and clinical trials of cognitive-sparing interventions in patients undergoing intracranial RT are often designed to detect changes in memory, specifically verbal memory.^4–8^

Despite the clinical focus on memory impairment, the underlying mechanistic brain injury that contributes to these memory deficits after RT is poorly understood. Injury to the hippocampus, a subcortical structure embedded deep within the temporal lobe and integral to episodic memory,^9^ is believed to be central to post-RT memory impairment. As a result, clinical trials of cognitive-sparing RT have focused *solely on avoidance of the hippocampi*, particularly the neurogenic stem cells in the subgranular zone (SGZ) of the dentate gyrus.^5,10^ However, the hippocampus does not subserve memory in isolation—it is only one component of a network of brain structures critical to memory.^11^ In addition to the hippocampus and other subcortical structures, the Papez circuit comprises several white matter (WM) tracts and connections, including the fornix, dorsal cingulum, and parahippocampal cingulum (PHC). Damage to these limbic tracts has been associated with memory deficits in patients with epilepsy, mild cognitive impairment, and cerebrovascular disease.^12–14^

Diffusion tensor imaging (DTI) and volumetric MRI are non-invasive, highly sensitive techniques that can be used to measure changes in WM integrity. Diffusion imaging biomarkers correlate with pathologic WM demyelination and axonal injury after radiation in vivo. High resolution volumetric imaging coupled with advanced image processing and automated parcellation techniques can be used to measure morphometry, microstructural integrity,and volumetric changes within the hippocampus as well as within discrete WM regions. These quantitative neuroimaging biomarkers are well-established and validated metrics used to quantify the microstructural integrity of white matter and subcortical structures in vivo, in a wide range of neurosciences studies and brain disease states.^15–19^ Only a few studies have explored RT-induced injury within individual limbic WM tracts and the impact on memory outcomes. Our previous work demonstrated that the fornix and cingulum showed considerable vulnerability to dose-dependent injury among WM tracts after RT.^20^ We also found that biomarkers of injury to medial temporal lobe superficial WM was associated with post-RT memory impairment.^21^ Another study found that diffusion biomarkers within the PHC was associated with post-RT verbal memory and verbal fluency in primary brain tumor patients.^22^ However, no studies to date have investigated the functional impact of RT effects on the Papez circuit and its integrity as a network, including the hippocampus and the efferent and afferent WM tracts that communicate with the hippocampus.

In this prospective study, we sought to explore biomarkers of integrity and morphometry within the bilateral hippocampi, as well as bilateral fornix, dorsal cingulum, and PHC, including the effects of RT dose and relationship between these biomarkers and verbal and visuospatial memory performance after brain RT. Such a “network” type approach allows us to better examine radiation effects on memory beyond the neurogenic stem cell hypothesis alone. We hypothesized that poorer microstructural integrity within the hippocampi, fornix, dorsal cingulum, and PHC, characterized by volume loss, higher mean diffusivity (MD), and reduced fractional anisotropy (FA), would correlate with worse memory performance. Further understanding of these effects will allow us to better understand the mechanistic underpinnings of memory function after brain RT, and to create more sophisticated memory preservation techniques in radiation therapy.

## Methods

### Study Participants

This prospective, observational clinical trial was approved by the UC San Diego Institutional Review Board. Written, informed consent was obtained from all patients. Adult patients with primary brain tumors receiving fractionated, partial-brain RT were eligible for enrollment. Inclusion criteria included age ≥ 18, Karnofsky performance status (KPS) ≥ 70, estimated life expectancy ≥ 1 year, and ability to undergo neurocognitive testing in English. Patients who received prior brain RT were excluded. Patient demographics, clinical, and treatment characteristics were prospectively recorded for all participants including sex, age, highest education level, ethnicity, race, baseline Karnofsky performance status (KPS), tumor location, radiation treatment modality, tumor histology, history of neurosurgery, history of seizures, receipt of chemotherapy, as well as receipt of corticosteroids or anti-epileptic drugs.

### Study Design

Patients underwent diffusion imaging and volumetric high-resolution MRI along with comprehensive neurocognitive testing (including verbal and visuospatial memory) prior to and 3, 6, and 12 months post-RT. For this study, we investigated brain regions which neuroanatomically subserve verbal and visuospatial memory: the hippocampus and its afferent and efferent WM tracts, including the fornix, dorsal cingulum, and parahippocampal cingulum (PHC). Study participants with any evidence of radiographic or clinical tumor progression or recurrence during the study period were removed from the analysis, as per protocol.

### Memory Assessments

Verbal and visuospatial learning and memory were evaluated at each time point using two robust, validated neuropsychological measures: the Hopkins Verbal Learning Test-Revised (HVLT-R) (a measure of verbal learning) and the Brief Visuospatial Memory Test-Revised (BVMT-R) (a measure of visuospatial memory and learning). Both HVLT-R and BVMT-R have Total Recall (HVLT-Total, BVMT-Total) and Delayed Recall (HVLT-Delayed, BVMT-Delayed) scores.^23,24^ All neurocognitive tests were administered or supervised by a licensed clinical neuropsychologist, one on one with the study subject.

### Imaging Acquisition

The imaging acquisition of high-resolution volumetric and diffusion-weighted MRIs for this study have been described in detail elsewhere.^25,26^ Briefly, imaging for all patients at each timepoint was acquired on a 3.0T 750 GE system (GE Healthcare, Milwaukee, Wisconsin) equipped with an 8-channel head coil. Sequences selected for the protocol included a 3D volumetric T1-weighted inversion recovery spoiled gradient echo sequence (echo time [TE]/repetition time [TR] = 2.8/6.5 ms; inversion time [TI] = 450 ms; flip angle = 8 °C; field of view [FOV] = 24 cm), a 3D FLAIR sequence (TE/TR = 125/6000 ms, TI = 1868 ms, FOV = 24 cm, matrix = 256 × 256, slice thickness = 1 mm), and a diffusion weighted imaging (DWI) sequence using a single-shot pulsed-field gradient spin EPI sequence (TE/TR = 96 ms/17 s; FOV = 24 cm, matrix = 128 × 128 × 48; inplane resolution 1.87 × 1.875; slice thickness = 2.5 mm; 48 slices) with b = 0, 500, 1500, and 4000 s/mm^2^, with 1, 6, 6, and 15 unique gradient directions for each b-value respectively and one average for each non-zero *b*-value. Two additional *b* = 0 volumes were acquired with either forward or reverse phase-encode polarity for use in nonlinear *B*_0_ distortion correction.^27^

### Image Processing

Preprocessing of imaging data was completed using in-house algorithms derived in MATLAB. We corrected for anatomical imaging distortions due to gradient nonlinearities,^28^ as well as diffusion scan spatial distortions caused by susceptibility and eddy currents.^27^ FA and MD maps were derived by fitting the DWI data from *b* values of 0, 500, and 1500 s/mm^2^ to a tensor. Within each voxel, an ellipsoid defined by three perpendicular axes (eigenvectors) approximated the diffusion process. FA is a unitless expression of the degree of directionality of diffusion that ranges from 0 to 1 and decreases with WM injury.^29^ MD is an average of the three eigenvalues expressed in µm^2^/ms that represents the average mobility of water molecules. MD increases with WM injury.^29^ Volume is reported here as cm^3^.

**Figure 1.**
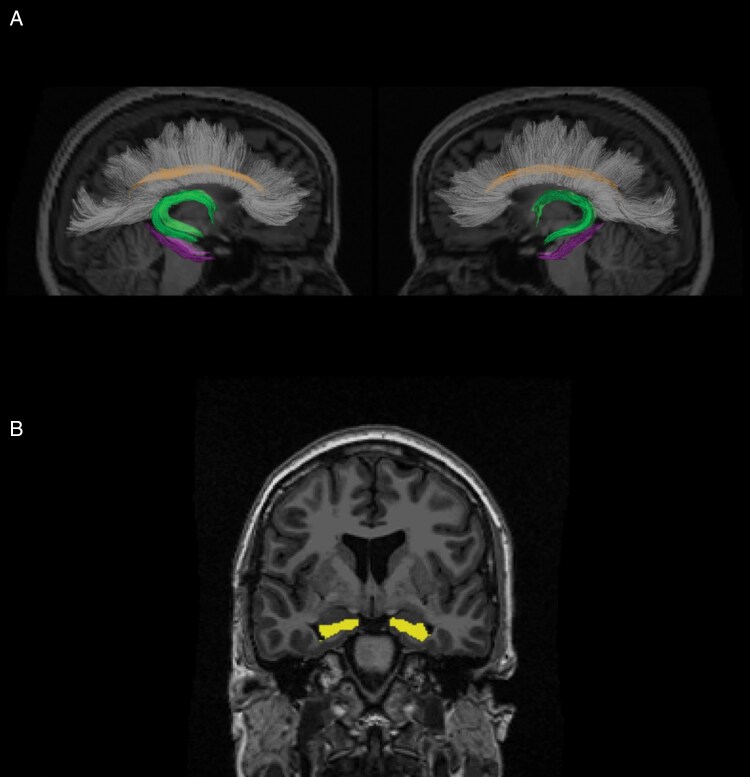
Memory network regions of interest (ROIs). (1A) Sagittal view displaying the fornix (green), dorsal cingulum (orange), and parahippocampal cingulum (PHC, purple). (1B) Frontal view displaying the hippocampus (yellow).

Selected regions of interest (ROIs) included the hippocampus and limbic WM tracts of the Papez circuit including the fornix, dorsal cingulum, and PHC (**Figure 1**). The hippocampus was segmented using the automated FreeSurfer processing pipeline (version 5.3;  https://surfer.nmr.mgh.harvard.edu/pub/dist/freesurfer/5.3.0/), available on the Neuroscience Gateway Portal.^30,31^ The DWI-derived maps and high-resolution volumetric MRI were co-registered and atlas-based white matter tractography was used to segment the DWI into WM tracts.^32^ Volume, FA, and MD were assessed in all WM ROIs, while volume alone was assessed in the hippocampus. FA and MD values were averaged across each WM ROI. A censoring mask was drawn manually slice by slice on each image at each timepoint to exclude tissue affected by tumor (non-enhancing and enhancing), surgical cavity or scarring, or edema, blinded to region of interest. The voxels within the censoring mask were excluded from the final ROI to avoid confounding by tumor and edema-related effects.^20^ Planning CT and RT dose maps were co-registered to the baseline T1 and DWI volumes and used to analyze average dose to each ROI.

### Reliable Change Indices

To analyze whether there were any acute and subacute changes in verbal and visuospatial memory at the individual-subject level, we calculated reliable change indices accounting for practice effects (RCI-PE) between baseline (pre-RT) and each post-RT time point score (3, 6, and 12 months). This method controls for practice effects and the measurement error of the test by adjusting a given individual’s raw change score using the mean practice effect of a reference group (ie, a test-retest normative sample)^33^ to produce a *z*-score. Thus, these scores allow a within-subject estimate of change. An RCI-PE score of 0 reflects no change from baseline, negative RCI-PE scores indicate decline from baseline, and positive scores signify improvement from baseline. One-sample *t*-tests evaluated significant change in RCI-PEs for each assessment from baseline to post-RT (*H*_0_ = 0).

### Statistical Analyses

We investigated the change in imaging biomarkers within each ROI over time using linear mixed-effects analyses. Models included time as a main effect, subject-specific random intercepts, and ROI volume, FA, or MD as the outcome. We also interrogated radiation dose-dependent changes on biomarkers of injury within ROIs over time using linear mixed effects analyses. These models assessed the relationship between imaging biomarkers and mean RT dose as a main effect. All models evaluating volume as an outcome included the percentage of ROI censored as a main effect to control for longitudinal censoring trends in volume measurements.^34^ Linear mixed-effects models are well suited for these longitudinal analyses since they account for within-subject correlation between repeated measures (given testing and imaging at several time points over time) and allow for incomplete outcome data.

Next, we evaluated imaging biomarkers of injury as predictors of memory scores over the entire study period using scores from each time point. Each linear mixed-effects model was fitted with either ROI volume, FA, or MD and time as main effects, a subject-specific random intercept, and raw neurocognitive score (verbal or visuospatial learning and memory) as the outcome. As above, all models including volume as a predictor additionally included the percentage of ROI censored as a main effect. These models are ideal for data analyses in cognitive neuroscience where within-participant data analyses are common. The subject-specific random intercept included in the models accounts for variation across study participants in their baseline neurocognitive testing scores and baseline imaging biomarkers. Time is a main effect, given that we have longitudinal, clearly defined end points over time. All linear mixed-effects models were fitted using the *lme4* package for R. Statistical significance was set at *α* = 0.05 for two-tailed tests. *P*-values were corrected for multiple comparisons using the adaptive Benjamini-Hochberg method to control the false discovery rate.^35^

## Results:

### Patient Characteristics

A total of 57 patients on trial who completed diffusion and volumetric MR imaging at each specified time point, and neurocognitive assessments at each time point during the study period were included in the present analyses. Demographic and clinical characteristics are shown in **Table 1**. I Median age was 45 years, with a slight majority of males (57%). Most patients had gliomas (63%) and the majority (77%) were treated with intensity-modulated RT or volumetric modulated arc photon therapy (IMRT/VMAT). Patient demographics, including age, receipt of chemotherapy, tumor type and histology, and receipt of anti-epileptic drugs were not significantly associated with any memory outcomes (*P* > .05). None of the study participants (*n* = 57) had radiographic or clinical tumor progression in the study period.

**Table 1:** Demographic and clinical characteristics of patient cohort (*n* = 57)

Characteristic	No. of Participants (%)
Median age, years (range)	45 (20-72)
Sex	
Male	33 (57%)
Female	24 (43%)
Ethnicity	
Non-Hispanic:	
Asian/Pacific Islander	3 (5%)
Black	1 (2%)
Middle eastern	0 (0%)
White	47 (82%)
Hispanic	7 (12%)
Highest education achieved, median (range)	16 (10-20)
High school	10 (18%)
College	28 (49%)
Graduate School	19 (33%)
Tumor Diagnosis	
High grade glioma	
IDH-WT glioblastoma, grade 4	12 (21%)
IDH-mutant astrocytoma, grade 4	4 (7%)
IDH-mutant astrocytoma, grade 3	9 (16%)
IDH-mutant oligodendroglioma, grade 3	3 (5%)
Glioneuronal tumor, grade 3	1 (2%)
Low Grade Glioma	
IDH-mutant astrocytoma, grade 2	1 (2%)
IDH-mutant oligodendroglioma, grade 2	5 (9%)
Pilocytic astrocytoma, grade 1	1 (2%)
Ependymoma, grade 2	1 (2%)
Meningioma	
Grade 1 meningioma	6 (11%)
Grade 2 meningioma	6 (11%)
Schwannoma	2 (4%)
Craniopharyngioma	2 (4%)
Pituitary adenoma	3 (5%)
Low grade chondrosarcoma	1 (2%)
Tumor side	
Left	28 (40%)
Right	25 (44%)
Central	4 (7%)
Tumor region	
Frontal	19 (33%)
Temporal	15 (26%)
Suprasellar	7 (12%)
Parietal	6 (11%)
Base of skull	3 (5%)
Cerebellar	3 (5%)
Cavernous sinus	2 (4%)
Sphenoid wing	2 (4%)
RT type	
IMRT/VMAT	44 (77%)
Proton	13 (23%)
Dose schedule	
59.4 Gy / 33 Fx	25 (44%)
54 Gy / 30 Fx	14 (25%)
60 Gy / 30 Fx	10 (18%)
50.4 Gy / 28 Fx	7 (12%)
70 Gy / 35 Fx^a^	1 (2%)
PTV Volume, cc	
Median (IQR)	141.74 (64.28 – 218.67)
Baseline KPS	
80	3 (5%)
90	42 (74%)
100	12 (21%)
Surgery	
GTR	14 (25%)
STR	33 (58%)
NTR	1 (2%)
Biopsy	4 (7%)
None	5 (9%)
Chemotherapy	36 (63%)
Concurrent + Adjuvant TMZ	28 (53%)
Concurrent + Adjuvant TMZ + Other^b^	3 (5%)
Adjuvant PCV only	5 (9%)
Seizures^c^	26 (46%)
AED^d^	34 (60%)
Steroids^d^	25 (44%)
Previously treated mood symptoms:	
Anxiety	7 (13%)
Depression	8 (14%)

Abbreviations: IDH, isocitrate dehydrogenase gene (mutant refers to mutation in IDH-1 or IDH-2; WT refers to wildtype or no IDH mutation); IMRT, intensity-modulated radiotherapy; VMAT, volumetric-modulated arc therapy; Fx, fractions; IQE, interquartile range; KPS, Karnofsky performance status; GTR, gross total resection; STR, sub-total resection; AED, anti-epileptic drug; TMZ, Temozolomide; PCV, Procarbazine, Lomustine, and Vincristine.

^a^ = Prescription dose to low grade chondrosarcoma.

^b^ = includes additional adjuvant therapy or clinical trials: Vaccine clinical trial (*n* = 1), Parp-inhibitor clinical trial (*n* = 1), adjuvant CCNU (*n* = 1).

^c^ = history of seizures or active seizures during study period.

^d^ = received during study period.

### Changes in Memory Function over Time

**Supplementary Table 1** shows the RCI-PEs for change in verbal and visuospatial memory performance between baseline and 3-, 6-, and 12-months post-RT. There was significant decline in group BVMT-R Total scores between baseline and 3 months (RCI-PE -0.844, *P* = 0.001), baseline and 6 months (RCI-PE -1.41, *P* < 0.001), and baseline and 12 months (RCI-PE −0.805, *P* < 0.001). Additionally, there was significant decline in HVLT Total scores from baseline to 12 months (RCI-PE −0.784, *P* = 0.002) and HVLT Delayed Recall scores from baseline to 12 months (RCI-PE -1.035, *P* = 0.001).

### Volume and DTI Biomarkers over Time

**Table 2** shows the beta estimates and *P*-values from linear mixed-effects analyses of changes in ROI volume, FA, and MD over time overall, as well as at specific post-RT timepoints. There was significant atrophy of the left fornix (*b* = −0.135 month*cm^3^, *P* < 0.001), left PHC (*b* = −0.014 month*cm^3^, *P* = 0.044), right hippocampus (*b* = −0.093 month*cm^3^, *P* = 0.020), right fornix (*b* = −0.130 month*cm^3^, *P* < 0.001), and right dorsal cingulum (*b* = −0.015 month*cm^3^, *P* = 0.010) overall and at specific timepoints. There were significant decreases in FA and increases in MD reflecting progressive white matter injury in the left and right fornix and dorsal cingulum at specific post-RT timepoints..

**Table 2.** Change in ROI volume and DTI biomarkers over time.

Outcome	Brain ROI		Overall^*^		3 Months		6 Months		12 Months	
			β coefficient (SE)	*P*-value	β coefficient (SE)	*P*-value	β coefficient (SE)	*P*-value	β coefficient (SE)	*P*-value
**Volume** (cm^3^/month)	Left	Hippocampus	−0.072 (0.032)	0.077	−0.041 (0.030)	0.167	−0.108 (0.044)	0.015*	−0.079 (0.046)	0.093
		Fornix	−0.135 (0.025)	<0.001*	−0.095 (0.025)	<0.001*	−0.132 (0.030)	<0.001*	−0.109 (0.033)	0.003*
		Dorsal Cingulum	−0.052 (0.019)	0.303	−0.055 (0.021)	0.012*	−0.053 (0.023)	0.022*	−0.042 (0.022)	0.0682
		PHC	−0.014 (0.009)	0.044*	0.001 (0.009)	0.950	−0.013 (0.012)	0.255	0.011 (0.011)	0.302
	Right	Hippocampus	−0.093 (0.033)	0.020*	−0.047 (0.033)	0.161	−0.089 (0.038)	0.023*	−0.049 (0.035)	0.176
		Fornix	−0.130 (0.026)	<0.001*	−0.098 (0.033)	0.003*	−0.129 (0.029)	<0.001*	−0.132 (0.028)	<0.001*
		Dorsal Cingulum	−0.015 (0.017)	0.010*	−0.026 (0.019)	0.174	−0.017 (0.018)	0.333	−0.043 (0.020)	0.035*
		PHC	−0.031 (0.014)	0.286	0.003 (0.013)	0.812	−0.031 (0.015)	**0.047**	−0.007 (0.014)	0.631
FA (1/month)	Left	Fornix	−0.008 (0.003)	0.052	−0.007 (0.004)	0.049	−0.008 (0.003)	0.010*	−0.010 (0.003)	<0.001*
		Dorsal Cingulum	−0.004 (0.003)	0.053	−0.006 (0.002)	0.005*	−0.005 (0.003)	0.129	−0.013 (0.003)	<0.001*
		PHC	−0.001 (0.005)	0.729	−0.003 (0.004)	0.540	−0.001 (0.005)	0.817	0.001 (0.003)	0.856
	Right	Fornix	−0.005 (0.004)	0.312	−0.005 (0.003)	0.108	−0.006 (0.005)	0.020*	−0.009 (0.003)	0.002*
		Dorsal Cingulum	−0.003 (0.003)	0.425	−0.003 (0.003)	0.312	−0.004 (0.004)	0.314	−0.010 (0.003)	0.004*
		PHC	0.0001 (0.004)	0.683	−0.004 (0.003)	0.241	−0.0003 (0.005)	0.958	−0.002 (0.003)	0.584
MD ((μm^2^/ms)/ month)*10^3	Left	Fornix	−0.006 (0.106)	0.337	0.016 (0.008)	0.061	0.007 (0.011)	0.528	0.021 (0.008)	0.016*
		Dorsal Cingulum	−0.010 (0.007)	0.288	−0.010 (0.003)	0.762	−0.011 (0.009)	0.244	0.00004 (0.006)	0.993
		PHC	−0.007 (0.010)	0.173	0.008 (0.008)	0.284	−0.008 (0.012)	0.512	0.006 (0.007)	0.427
	Right	Fornix	−0.001 (0.011)	0.137	0.019 (0.007)	0.007*	−0.003 (0.015)	0.859	0.029 (0.009)	0.003*
		Dorsal Cingulum	−0.001 (0.006)	0.307	−0.010 (0.003)	0.735	−0.009 (0.008)	0.253	−0.002 (0.004)	0.707
		PHC	−0.008 (0.007)	0.306	0.004 (0.003)	0.140	−0.008 (0.009)	0.424	0.0002 (0.003)	0.914

Significant results (*P* < 0.05) are shown in bold, with asterisks (*) indicating results that remained significant after correcting for multiple comparisons.

Overall change over time across all study points (3, 6 and 12 months).

Abbreviations: ROI, region of interest; FA, fractional anisotropy; MD, mean diffusivity; PHC, parahippocampal cingulum.

### Mean Dose as a Predictor of Injury Biomarkers

Mean dose to each ROI after censoring tumor, cavity, and edema is shown in **Supplementary Table S2**. There was no significant difference between mean doses to right and left ROIs.

Beta estimates and *P*-values from linear mixed-effects analyses of the association between mean dose to each ROI and volume, FA, and MD are shown in **Table 3**. The higher dose was significantly associated with greater atrophy of the left hippocampus (*b* = −0.0159 Gy/(month*cm^3^)*10^3^, *P* < 0.001), right hippocampus (*b* = −0.0229 Gy/(month*cm^3^)*10^3^, *P* < 0.001), and right fornix (*b* = −−0.009 Gy/(month*cm^3^)*10^3^, *P* = 0.028). Higher dose also predicted reduced FA within the right PHC (*b* = −0.0005 Gy/month*10^3^, *P* = 0.042). Higher dose to the right PHC was significantly associated with greater MD (*b* = 0.00168 Gy/[month*μm^2^/ms]*10^6^, *P* = 0.028).

**Table 3.** Association between mean RT dose and imaging biomarkers within each ROI.

Outcome	Brain ROI		β coefficient (SE)	*P*-value
**Volume** ^†^ Gy/(month*cm^3^)*10^3^	Left	Hippocampus	−15.9 (4.4)	<0.001*
		WM tracts:		
		Fornix	7.6 (4.0)	*0.063*
		Dorsal Cingulum	−2.7 (2.8)	*0.340*
		PHC	−0.4 (1.1)	*0.753*
	Right	Hippocampus	−22.9 (3.9)	<0.001*
		WM tracts:		
		Fornix	−9.0 (4.0)	0.028*
		Dorsal Cingulum	−2.9 (2.9)	*0.324*
		PHC	−1.6 (1.2)	*0.169*
**FA** Gy/month*10^3^	Left	Hippocampus	-	-
		WM tracts:		
		Fornix	−8.7 (24.0)	*0.716*
		Dorsal Cingulum	−51.1 (39.2)	*0.199*
		PHC	−11.8 (32.5)	*0.729*
	Right	Hippocampus	-	-
		WM tracts:		
		Fornix	−45.0 (22.3)	***0.049***
		Dorsal Cingulum	−24.7 (33.2)	*0.449*
		PHC	−56.3 (27.3)	0.042*
**MD** Gy/(month*μm^2^/ms)*10^6^	Left	Hippocampus	-	-
		WM tracts:		
		Fornix	0.46 (0.84)	*0.584*
		Dorsal Cingulum	0.67 (0.48)	*0.162*
		PHC	0.78 (0.71)	*0.275*
	Right	Hippocampus	-	-
		WM tracts:		
		Fornix	0.96 (0.77)	*0.215*
		Dorsal Cingulum	0.40 (0.50)	*0.162*
		PHC	1.68 (0.75)	0.028*

Significant results (*P* < 0.05) are shown in bold and those that remained significant after correcting for multiple comparisons are bolded with asterisks.

Abbreviations: ROI, region of interest; FA, fractional anisotropy; MD, mean diffusivity; PHC, parahippocampal cingulum;.

^†^Models control for percent volume censored.

### Biomarkers of Injury as Longitudinal Memory Predictors

The associations between imaging biomarkers of microstructural integrity within ROIs and memory performance assessed at the same timepoint are shown in **Table 4**. There were no significant associations between right or left hippocampal volumes and verbal or visuospatial memory scores. Greater volume of the left fornix was associated with better BVMT-Total (*b* = 1.61 points/(month*cm^3^), *P* = 0.019) and BVMT-Delayed (*b* = 0.481 points/(month*cm^3^), *P* = 0.026) scores. Higher FA (*b* = 35.5 points/month, *P* = 0.011) and lower MD (*b* = −11.58 points/(month*μm^2^/ms), *P* = 0.01) within the left fornix correlated with better BVMT-Total performance. Greater FA within the left dorsal cingulum was associated with higher HVLT-Total scores (*b* = 21.5 points/month, *P* = 0.038), and higher volume of the left PHC correlated with better BVMT-Delayed scores (*b* = 3.18 points/(month*cm^3^), *P* = 0.04). Within the right fornix, greater volume (*b* = 2.55 points/(month*cm^3^), *P* = 0.019) and FA (*b* = 29.0 points/month, *P* = 0.02) as well as lower MD (*b* = −12.28 points/(month*μm^2^/ms)/10^4^, *P* = 0.007) were associated with better BVMT-Total performance. Within the right PHC, higher FA (*b* = 11.9 points/month, *P* = 0.039) correlated with better HVLT-Delayed scores, while lower MD was associated with higher BVMT-Total (*b* = −16.21 points/(month*μm^2^/ms)/10^4^, *P* = 0.01) scores.

**Table 4.** Association between imaging biomarkers of injury and memory scores within each ROI.

Brain ROI		Biomarker	BVMT-T		BVMT-D		HVLT-T		HVLT-D	
			β coefficient (SE)	*P*-value	β coefficient (SE)	*P*-value	β coefficient (SE)	*P*-value	β coefficient (SE)	*P*-value
**Left sided:**	HC	Volume^†^	0.906 (0.915)	0.331	0.355 (0.376)	0.347	−0.197 (0.821)	0.810	−0.190 (0.438)	0.666
	WM tracts:									
	Fornix	Volume^†^	1.61 (1.07)	0.019*	0.481 (0.418)	0.026*	−0.024 (0.942)	0.979	−0.060 (0.513)	0.907
		FA	0.35 (0.14)	0.011*	0.030 (0.061)	0.636	0.15 (0.13)	0.250	0.043 (0.067)	0.535
		MD	−11.58 (4.44)	0.010*	−2.57 (1.93)	0.185	−0.23 (4.16)	0.957	0.82 (2.21)	0.711
	Dorsal Cingulum	Volume^†^	1.62 (1.48)	0.276	0.477 (0.578)	0.411	−0.680 (1.35)	0.613	−0.732 (0.736)	0.320
		FA	0.11 (0.12)	0.351	0.075 (0.044)	0.092	0.21 (0.10)	0.038*	0.077 (0.056)	0.173
		MD	−6.76 (8.29)	0.421	−3.68 (3.57)	0.310	3.25 (7.67)	0.677	3.69 (4.07)	0.371
	PHC	Volume^†^	5.60 (3.79)	0.143	3.18 (1.54)	0.040*	1.40 (3.43)	0.68	−0.620 (1.81)	0.732
		FA	0.12 (0.10)	0.222	0.031 (0.045)	0.503	0.020 (0.093)	0.833	−0.028 (0.049)	0.566
		MD	−7.93 (5.53)	0.153	−3.76 (2.41)	0.123	−2.12 (5.16)	0.684	0.778 (2.72)	0.777
**Right sided:**	HC	Volume^†^	1.03 (0.880)	0.246	0.371 (0.359)	0.307	0.879 (0.832)	0.291	−0.042 (0.432)	0.923
	WM tracts:									
	Fornix	Volume^†^	2.55 (1.07)	0.019*	0.837 (0.417)	0.051	−1.30 (0.981)	0.186	−0.674 (0.536)	0.210
		FA	0.29 (0.12)	0.020*	0.068 (0.055)	0.217	0.11 (0.11)	0.293	0.029 (0.060)	0.633
		MD	−12.28 (4.51)	0.007*	−3.20 (1.99)	0.109	2.13 (4.27)	0.619	0.990 (2.27)	0.663
	Dorsal Cingulum	Volume^†^	0.622 (1.54)	0.686	−0.190 (0.592)	0.749	0.762 (1.37)	0.580	−0.150 (0.756)	0.843
		FA	0.0007(0.12)	0.96	0.049 (0.050)	0.347	0.042 (0.10)	0.695	0.032 (0.058)	0.580
		MD	−13.57 (8.70)	0.124	−4.98 (3.73)	0.187	1.46 (7.98)	0.856	0.365 (4.25)	0.932
	PHC	Volume^†^	3.34 (3.14)	0.289	1.03 (1.30)	0.428	0.815 (2.80)	0.771	1.88 (1.51)	0.215
		FA	0.19 (0.12)	0.107	0.021 (0.053)	0.692	0.20 (0.11)	0.065	0.12 (0.060)	0.039*
		MD	−16.21 (6.28)	0.010*	−2.37 (2.63)	0.367	−16.10 (7.99)	**0.046**	−7.68 (4.29)	0.078

Significant results (*P* < 0.05) are shown in bold, with asterisks (*) indicating results that remained significant after correcting for multiple comparisons.

Units for the β coefficient are as follows: volume, points/(month*cm^3^); FA, points/month; MD, points/(month*μm^2^/ms)/10^4^.

Abbreviations: ROI, region of interest; FA, fractional anisotropy; MD, mean diffusivity; WM, white matter; HC, hippocampus; PHC, parahippocampal cingulum.

^†^Models controlled for percent volume censored.

## Discussion

Cognitive-sparing techniques for memory preservation must be informed by an understanding of the mechanistic and neuroanatomic injury underlying memory loss, including injury to broader memory networks, beyond the hippocampus. We present the first prospective analysis of longitudinal changes in memory performance after RT including in vivo biomarkers of microstructural integrity within the limbic networks of the Papez circuit, including the hippocampus as well as its afferent and efferent white matter connections. We identified time- and dose-dependent microstructural injury within the hippocampus, fornix, dorsal cingulum, and PHC after RT. Moreover, we found that WM damage within the fornix, dorsal cingulum, and PHC correlated with poorer memory performance, while hippocampal atrophy did not. Current memory-sparing treatment strategies are based solely on limiting RT exposure to the hippocampus. Our results demonstrate that optimal post-treatment memory preservation should not be limited to hippocampal-sparing techniques alone but should also focus on minimizing damage to efferent and afferent memory-associated WM tracts.

To our knowledge, this is the first study to demonstrate the functional consequences of RT-mediated white matter injury to the fornix. The fornix, a C-shaped bundle of white matter, comprises the major afferent and efferent tracts from the hippocampus.^15^ Specifically, we found that loss of microstructural integrity (bilateral fornix atrophy, reduced FA, and greater MD), correlated with worse visuospatial memory. Microstructural integrity of the fornix has been shown to correlate with memory outcomes in a variety of populations. In a study of healthy aging patients, fornix damage was shown to be a predictor of incipient cognitive decline, while hippocampal atrophy was not.^16^ Another investigation found that decreased fornix FA was one of the earliest MRI abnormalities observed in cognitively normal individuals at increased risk for developing Alzheimer’s disease (AD).^17^ Damage to the fornix has also been shown to correlate with hippocampal atrophy in these populations.^17,18^ Pelletier et al. suggest that fornix injury precedes and may contribute to hippocampal atrophy via a loss of connections between the two structures.^18^ Thus, the fornix comprises a critical node within the Papez circuit, and its integrity may influence that of other structures within the memory network. Interestingly, our study revealed associations between fornix integrity and visuospatial but not verbal memory performance. These findings were echoed in a recent preliminary trial of patients with temporal lobe epilepsy (TLE), in which theta burst stimulation of the fornix led to improved visual-spatial memory rather than verbal memory.^36^ Indeed, transection of the fornix has long been known to yield impaired navigational learning in animals. Current hippocampal-sparing regimens have demonstrated verbal memory preservation after treatment.^4,10^ Our results suggest that the incorporation of fornix avoidance into these plans may confer additional (i.e. visuospatial) memory benefits.

We also identified associations between PHC and dorsal cingulum integrity and memory. The PHC, located at the mediodorsal end of the cingulum, is an afferent tract to the hippocampus.^37^ Prior studies have uncovered associations between PHC injury and memory in in patients with TLE^38^ and AD.^17^ Only one prior study has examined the functional correlates of RT-mediated PHC injury, which demonstrated a relationship between PHC damage and verbal memory and fluency.^22^ Of note, we found that biomarkers of PHC microstructural integrity was associated with both verbal and visuospatial memory. Specifically, left PHC volume loss correlated with poorer visuospatial memory, while right PHC WM injury was associated with both poorer visuospatial and verbal memory scores. In general, verbal memory is thought to be linked to the left medial temporal lobe (MTL), while visuospatial memory is typically associated with right^39^ or bilateral MTL functioning.^40^ Our results suggest that, though dominant, the bilateral cerebral hemispheres are not exclusive to specific memory modes. *Memory-sparing treatment plans should therefore account for bilateral memory structures*. In the dorsal cingulum, we found that left-sided WM damage was associated with poorer verbal memory performance. The dorsal cingulum connects the parietal, frontal, and temporal lobes, and also serves as an afferent tract to the hippocampus.^37^ Damage to the cingulum has been linked to verbal memory deficits in older adults^41^ and patients with small vessel disease.^14^ Our group also previously demonstrated an association between post-RT verbal ability and integrity of the superficial WM beneath the anterior cingulate gyrus, which includes the dorsal part of the cingulum bundle.^42^ The results from the present study confirm the role of the PHC and dorsal cingulum in post-RT memory function. Dose constraints to these structures may therefore contribute to further memory preservation after treatment.

Notably, hippocampal volume was not associated with verbal or visuospatial memory performance among brain tumor patients in our cohort. This finding aligns with other studies such as the secondary analysis of RTOG 0933, which found no association between hippocampal volume change and verbal memory change among patients receiving hippocampal avoidance whole brain RT,^7^ or another small study among 15 patients with high grade gliomas that found no association between hippocampal thickness on structural MRI and objective memory performance.^8^ However, it does differ from our previous work,^42^ where we found that right hippocampal atrophy correlated with worse visuospatial memory performance in a subset of this cohort (22 patients). This discrepancy may be attributable to several factors. Memory is multi-faceted, comprising episodic, semantic, procedural, and working memory,^43^ and the hippocampus subserves several of these forms of memory.^9,44^ Thus, the HVLT and BVMT, which are commonly used to assess episodic memory function after brain tumor treatment in clinical trials,^5,10,45^ may not capture the full range of memory effects from hippocampal RT exposure. In addition, the hippocampus alone, without considering its afferent and efferent WM tracts, may have only marginal effects on episodic memory specifically. This idea is in line with recent studies in neurosurgical populations that demonstrated a stronger contribution of medial temporal white matter tracts than hippocampal volume in predicting post-surgical memory decline.^46,47^

**Figure 2.**
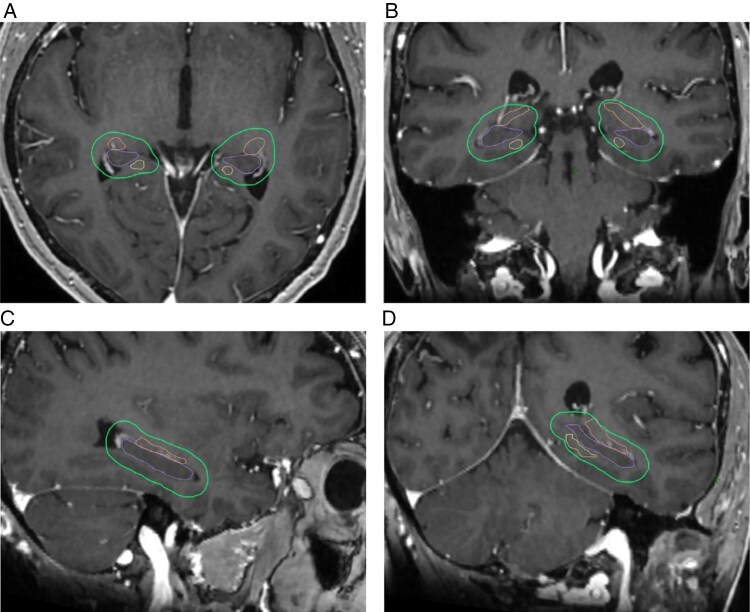
MRI of a brain with the anatomical borders of the hippocampus (purple), fornix (orange), and parahippocampal cingulum (PHC, yellow) with a 5 mm margin (green) in an axial (2A), coronal (2B), sagittal (2C), and oblique (2D) view.

Indeed, the evidence for memory preservation specifically with hippocampal avoidance is mixed. In a recent randomized controlled trial of patients with small cell lung cancer, there was no difference in the probability of verbal memory decline between patients undergoing prophylactic cranial irradiation with or without hippocampus avoidance.^45^The practice-changing NRG CC-001 trial revealed that the risk of cognitive decline was significantly lower among patients with brain metastases receiving hippocampal-sparing compared with non-hippocampal-sparing whole-brain RT and memantine.^10^ Importantly, however, this trial was powered based on the overall cognitive deterioration rate between treatment arms rather than the rate of memory decline specifically. Current hippocampal-sparing treatment protocols are based on avoidance of stem cell regions within the hippocampus, including the subgranular and subventricular zones.^5^ It is possible that hippocampal avoidance confers cognitive protection not specific to memory, mediated by avoidance of these neural stem cells. Furthermore, it may be that the cognitive protective benefits of hippocampal avoidance are not mediated by the prevention of atrophy. However, it is also likely that the hippocampal avoidance area in treatment planning may include portions of the critical medial temporal lobe white matter pathways that we have shown are most sensitive and specific. **Figure 2** shows an MRI example where the hippocampus, fornix, and PHC are segmented, with a 5mm margin around the hippocampus. This 5 mm hippocampal avoidance zone would be contoured and used for dose avoidance per the CC-001 trial. Note that the 5 mm margin of the hippocampus covers a substantial portion of both the fornix and the PHC, suggesting that “hippocampal” sparing may in fact be additionally avoiding the hippocampus’ major afferent and efferent WM tracts.

We also uncovered time- and dose-dependent atrophy to the hippocampi and microstructural injury to memory-associated WM tracts. Specifically, higher RT doses correlated with bilateral hippocampal atrophy as well as right fornix and right PHC microstructural WM injury. We previously identified RT-dose dependent hippocampal atrophy among 52 patients undergoing partial-brain RT.^48^ The effects of RT on WM are well documented,^49^ and late-manifesting WM injury (> 6 months post-RT) is believed to underlie much of the cognitive decline seen after treatment. Our findings parallel those from our prior work,^20^ where we found the fornix and cingulum to be the most sensitive to RT dose out of 21 WM tracts analyzed. Two similar studies of patients undergoing whole-brain RT,^19,50^ also identified the greatest changes in WM diffusion metrics within the fornix and cingulum after treatment. Of note, we did not identify any dose-dependent changes within the dorsal cingulum. This may reflect the lower sensitivity of this region to RT. These findings are in alignment with a prior study examining radiation sensitivity within cingulum regions,^19^ in which the authors found significant decreases in FA and increases in MD in the posterior but not superior portion of the cingulum between pre- and post-RT. Ultimately, our results indicate that critical memory structures beyond the hippocampus alone are sensitive to RT dose. Memory-sparing treatment protocols should therefore also include dose constraints for the fornix and PHC to mitigate RT-associated injury.

This study had several limitations. Our selected ROIs were parcellated using auto-segmentation programs *FreeSurfer* and *AtlasTrack*.^31,32^ While this eliminated inter-operator variability, these programs were developed using healthy controls without tumor-related alterations in their neuroanatomy. Nevertheless, both programs have been well validated and used in prior studies of patients with brain tumors.^21,34,45^ Additionally, our image processing procedures utilize high resolution input imaging data, all from the same scanner, providing internal validity. Segmentations at each timepoint for each patient were inspected slice-by-slice to manually censor out tumor, surgical cavities, and edema.^20,48^ Our sample size is relatively small, which limited the power of our study; however, it is larger than other studies assessing imaging biomarkers of memory in patients with brain tumors.^22,50^ Our dataset is also rich in prospectively measured longitudinal imaging and cognitive outcomes at pre-defined time points, and all neurocognitive testing was performed or supervised by a board-certified neuropsychologist. Tumor histology within our cohort was heterogeneous, and some patients received chemotherapy along with RT. Our study was not powered to analyze effects from chemotherapy or anti-epileptic drugs, and receipt of these treatments was not associated with memory outcomes. Future studies in different primary brain tumor cohorts may assess how chemotherapy may modulate radiation effects on imaging and neurocognitive outcomes. While we did censor all areas of tumor, edema and surgical scar from all imaging analyses, it is possible that in glioma patients, there could be tumor cells beyond this that are not visible on brain MRI which may affect imaging biomarkers. Yet, it is important to note that our analyses include bilateral structures, including the contralateral side from the tumor.

In conclusion, we present the first study to examine the effects of RT on the hippocampus and the major WM tracts (e.g. efferent and afferent connections) comprising the MTL memory network. We found that RT-associated loss of microstructural integrity within the fornix, dorsal cingulum, and PHC were associated with worse verbal and visuospatial learning and memory, while hippocampal atrophy was not. Our results suggest that hippocampal avoidance alone may not be sufficient for preventing memory decline after RT. Future cognitive-sparing efforts should expand their focus to the WM tracts of the Papez circuit to optimally preserve memory after radiation treatment.

## Supplementary material

Supplementary material is available online at *Neuro-Oncology* (https://academic.oup.com/neuro-oncology).

noaf144_Supplementary_Table_S1

noaf144_Supplementary_Table_S2

## Data Availability

Research data are stored in an institutional repository and anonymized data will be shared upon request to the corresponding author.
