# Supplementary material for: Beyond the hippocampus: Limbic white matter injury implicated in post-radiation memory performance in primary brain tumor patients
Source: Neuro Oncol. 2025 Jun 13;27(10):2647–60. doi: 10.1093/neuonc/noaf144 (PMC12833530; doi:10.1093/neuonc/noaf144)
Supplement: noaf144_Supplementary_Table_S1 [file noaf144_supplementary_table_s1.docx]

Supplemental Table 1: RCI-PEs for change in memory performance between baseline and 3-, 6-, and 12-months.

|  | 3 Months (p-value) | 6 Months (p-value) | 12 Months (p-value) |
| --- | --- | --- | --- |
| BVMT-Total | **-0.844 (0.001)** | **-1.407 (<0.001)** | **-0.805 (<0.001)** |
| BVMT-Delayed | 0 (0.5) | -0.382 (0.174) | -0.088 (0.419) |
| HVLT-Total | -0.351 (0.090) | -0.099 (0.342) | **-0.784 (0.002)** |
| HVLT-Delayed | -0.212 (0.203) | -0.106 (0.352) | **-1.035 (0.001)** |
| Abbreviations: RCI-PE, reliable change indices adjusted for practice effects; BVMT, Brief Visuospatial Memory Test; HVLT, Hopkins Verbal Learning Test | | | |
