# Supplementary material for: Beyond the hippocampus: Limbic white matter injury implicated in post-radiation memory performance in primary brain tumor patients
Source: Neuro Oncol. 2025 Jun 13;27(10):2647–60. doi: 10.1093/neuonc/noaf144 (PMC12833530; doi:10.1093/neuonc/noaf144)
Supplement: noaf144_Supplementary_Table_S2 [file noaf144_supplementary_table_s2.docx]

Supplemental Table 2: Mean dose in Gray to each ROI

|  | Hippocampus | Fornix | Dorsal Cingulum | PHC |
| --- | --- | --- | --- | --- |
| Left | 19.8 | 21.6 | 21.1 | 19.3 |
| Right | 18.1 | 20.6 | 21.2 | 17.6 |
| p-value | 0.154 | 0.317 | 0.928 | 0.158 |

Abbreviations: ROI, region of interest; PHC, parahippocampal cingulum
